# Eating habits and sleep patterns of adolescents with depression symptoms in Mumbai, India

**DOI:** 10.1111/mcn.12998

**Published:** 2020-12-21

**Authors:** Panchali Moitra, Jagmeet Madan, Nida I. Shaikh

**Affiliations:** ^1^ Department of Food, Nutrition & Dietetics, Sir Vithaldas Thackersey College of Home Science (Autonomous) SNDT Women University Mumbai India; ^2^ Department of Nutrition, Byrdine F Lewis College of Nursing & Health Professionals Georgia State University Atlanta Georgia USA

**Keywords:** adolescent, depression, eating habits, mental health, overweight, sleep pattern

## Abstract

Adolescents with depression engage in unhealthy eating habits and irregular sleep patterns and are often at an increased risk for weight‐related problems. Improvement in these lifestyle behaviours may help to prevent depression, but knowledge about the associations between depression, sleep, eating habits and body weight among adolescents in India is limited. This cross‐sectional study investigated the prevalence of depression and its association with sleep patterns, eating habits and body weight status among a convenience sample of 527 adolescents, ages 10–17 years in Mumbai, India. Participants completed a survey on sleep patterns such as sleep duration, daytime sleepiness and sleep problems and eating habits such as frequency of breakfast consumption, eating family meals and eating out. Depression was assessed using the Patient Health Questionnaire modified for Adolescents (PHQ‐A). Anthropometric measurements were also taken. Within this sample, 25% had moderate to severe depression (PHQ‐A ≥ 10) and 46% reported sleeping less than 6 h > thrice a week. Adolescents with moderate to severe depression had significantly higher body mass index than those with minimal depression (26.2 ± 6.6 vs. 20.2 ± 4.8 kg/m^2^). The odds of having clinically significant depression (PHQ‐A ≥ 10) was 4.5 times higher in adolescents who had family meals ≤ once a week, 1.6 times higher among those who were sleeping <6 h and 2.3 times higher among participants having trouble falling to sleep more than thrice a week. The findings indicated that a significant proportion of adolescents had depression symptoms; improving sleep and eating habits may present potential targets for interventions.

**Key messages**

- Adolescents often engage in poor lifestyle behaviours such as unhealthy eating habits and sleep insufficiency; these factors may predict depressive symptoms in adolescents.
- The study observed that a substantial proportion of adolescents suffered from depression and had sleep‐related problems and unhealthy eating habits. A significant difference was observed in the prevalence of depression between older adolescents, ages 16–17 years, and younger adolescents, ages <15 years, and between those who slept less than 6 h and those who reported sleeping ≥7 h more than thrice a week.
- Frequency of having family meals together, inadequate sleep duration and poor sleep quality emerged as important factors associated with depressive symptoms, thereby accentuating a need to foster better sleep and eating habits for improved mental well‐being among adolescents.

## 1 INTRODUCTION

Depressive disorders are common mental health problems in adolescents with an estimated prevalence of 10%–20% worldwide, according to a recent World Health Organization report on adolescent development (World Health Organization, 2019). Clinical studies report adolescent depression to be a chronic condition with high risk of recurrence in the future (Costello, Mustillo, Erkanli, Keeler, & Angold, 2003; Dunn & Goodyer, 2006; Maughan, Collishaw, & Stringaris, 2013), and several epidemiological studies have established associations between depression in adolescence and increased behavioural problems, impaired social functioning, poor academic performance, drug and alcohol abuse related behaviours, obesity and risk to suicide deaths (Costello et al., 2003; Erskine et al., 2017; Maughan et al., 2013; Thapar, Collishaw, Pine, & Thapar, 2012; Weissman et al., 1999). Addressing depression among adolescents, a socially and demographically vulnerable group, is therefore of major public health importance.

Although global data on the prevalence of depression among adolescents is available, studies that have evaluated the prevalence and severity of depressive symptoms among children and adolescents in low‐ and middle‐income countries such as India are limited (Grover, Raju, Sharma, & Shah, 2019). The few studies that have been conducted in India have either focused on the prevalence of depression among older adolescents, ages 16–17 years (Daryanavard et al., 2011; Jha et al., 2017; Saluja et al., 2004; Victor & Karunakaran, 2018), or younger adolescents, ages 13–15 years (Aradhya, 2013; M. M. Singh, Gupta, & Grover, 2017). Given that half of all mental health problems first emerge in late childhood and early adolescence (Kessler et al., 2007) and that a significant proportion of adolescent depression stays underdiagnosed (World Health Organization, 2019; Thapar et al., 2012), it is imperative that we study the prevalence of depression across the entire spectrum of adolescence, ages 10–17 years, and also the associated lifestyle behaviours that may exacerbate the severity of depression in this vulnerable age. Early identification of depression symptoms can provide opportunities to intervene, promote emotional and mental well‐being and prevent future risk to depression related negative outcomes among adolescents.

The transition from childhood to adolescence is frequently accompanied with an increase in poor lifestyle behaviours such as unhealthy eating habits and irregular sleep patterns. Sleep deprivation stems from early school timings, longer school hours, late night activities, disturbed sleep–wake schedule and lesser daytime napping opportunities; disturbed sleep can result in deficits in cognitive functioning, impaired mood and poor interpersonal social relationships (Baglioni et al., 2011). Concurrently, studies have observed that adolescents with depression experience significantly shorter sleep duration and insomnia as compared with those without depression, showing a potential bidirectional association between sleep disturbances and depression in adolescents (Alvaro, Roberts, & Harris, 2013; Roberts & Duong, 2014). Sleep problems have been studied among adolescents in India; a recent study conducted among adolescents ages 10–17 years in southern India reported that three‐fourths of adolescents were sleeping less than 8 h (Murugesan, Karthigeyan, Selvagandhi, & Gopichandran, 2018). Another study of 1000 school‐going adolescents, ages 15–17 years, in central India observed that the sleep quality deteriorated with increasing age (Saxena, Koreti, & Gaur, 2016) and a cross‐sectional study of sleep patterns in 11–15 year olds in Delhi showed the prevalence of sleep deprivation to be as high as 92.5% (R. Singh, Suri, Sharma, Suri, & Adhikari, 2018). Considering that sleep‐deprived adolescents are more likely to have behavioural problems (Gregory & O'Connor, 2002; Roberts & Duong, 2014; Yen, Ko, Yen, & Cheng, 2008) and that a significant number of adolescents in India do not obtain adequate sleep, it is important that the association between sleep patterns and depressive symptoms is understood.

In addition to sleep‐related issues, adolescence is a life stage that is marked by increasing autonomy in making food choices. Eating habits are identified as important predictors of mental health and well‐being (Sanchez‐Villegas & Martínez‐González, 2013; Wattick, Hagedorn, & Olfert, 2018). Evidence from longitudinal studies indicate that eating behaviours are inversely associated with the risk of depression among adolescents (Bamber, Stokes, & Stephen, 2007; Lang, Beglinger, Schweinfurth, Walter, & Borgwardt, 2015), though the associations between consumption of specific nutrients or specific foods and depression remain unclear. A systematic review of 20 studies evaluating the association between diet and depression in adolescents found no significant association between the consumption of fruits and vegetables and moods among adolescents (Khalid, Williams, & Reynolds, 2016), but found that depression was associated with ‘unhealthy’ eating behaviours such as frequent takeaways or eating away from home (Faith, Matz, & Jorge, 2002; Quirk et al., 2013) and skipping meals or breakfast (Lee et al., 2017). A consistent inverse association was reported between frequency of family dinners and depression; the relationship remained significant after adjusting for demographic and familial variables (Fulkerson et al., 2006), indicating the importance of family mealtimes as a strategy to promote adolescent mental health. Unhealthy eating behaviours such as missing breakfasts, overindulgence in fast foods while eating out and lack of family mealtimes have been reported among adolescents in several Indian studies (Beena, Idris, Savita, Reema, & Ashutosh, 2013; Faith et al., 2002; Jayawardena, Ranasinghe, Wijayabandara, Hills, & Misra, 2016; Mehta et al., 2014; Rathi, Riddell, & Worsley, 2018); thus, dietary habits can work as potential targets of public health interventions to decrease the burden of depression in adolescents in India.

Besides issues with sleep patterns and eating habits, there is substantial evidence of a dual burden of malnutrition in adolescence, which is believed to be worsened by depression (Jorm et al., 2003; Shin & Shin, 2008). A longitudinal study conducted to assess the association between overweight status and depression symptoms in a sample of adolescents, ages 11 to 21 years, in United States found body weight to be significantly associated with depressive symptoms for girls, after adjusting for exercise and sociodemographic characteristics (Needham & Crosnoe, 2005). Another study that evaluated the relationships between the body mass index (BMIs), body weight perception and depressed mood among Korean adolescents found that a low BMI and perceiving oneself as underweight were related to a depressed mood among adolescent boys. However, for girls, both low and high BMI were negatively related to depressed mood (Lim & Kim, 2017). The findings from these studies highlight the significance of body weight status as a determinant of depression symptoms and that adolescents who are not at their normal weight, both underweight and overweight, may be at an increased risk for depression. The potential associations between body weight status and depressive symptoms have not been explored among Indian adolescents.

To our knowledge, most of the current evidence on the association between lifestyle behaviours such as sleep patterns, eating habits and weight status among adolescents and depression symptoms are limited to studies from western settings including Europe, United States, and Australia. This study was conducted in a major urban city of a low‐ and middle‐income country, namely, Mumbai in India, to determine the prevalence of depression among 10‐ to 17‐year‐old adolescents and to assess sleep patterns, eating habits and body weight status. Our secondary objective was to investigate the association between depression, sleep, eating habits and body weight status among adolescents.

## 2 METHODOLOGY

**FIGURE 1:**
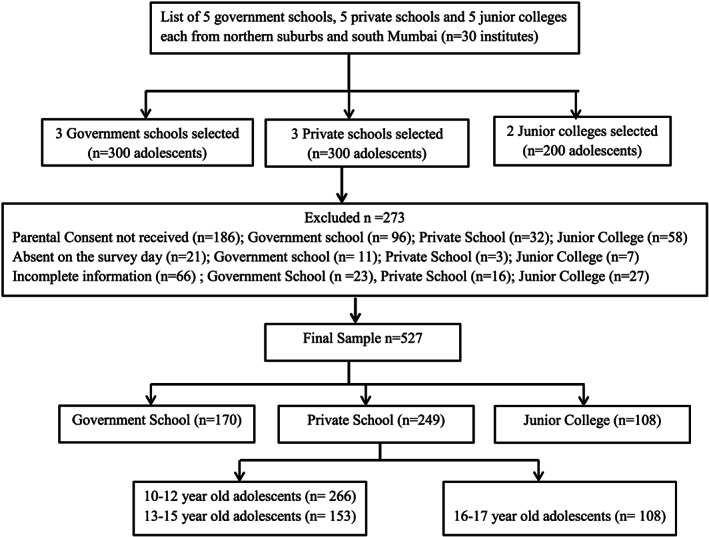
Method for selection of study sites and study sample

This cross‐sectional study was conducted among adolescents between the age group of 10 to 17 years in the city of Mumbai, western India, from January 2018 to February 2019. Using a purposive sampling method, we selected schools and junior colleges (for adolescents, ages 16–17 years) from southern and northern suburbs of Mumbai. We invited five private schools, five government schools and five junior colleges from each of these suburbs to participate in our study. The first three private schools, three government schools and two junior colleges that provided permission were selected as study sites. A class from Grade 5 to Grade 9 from each of the six schools and Grade 11 from each of the two junior colleges were selected at random by the school administration and college supervisor as the participating classes for the study. From each of these classes, we selected 20 students using random roll numbers from the class registers. The study protocol was verbally explained in detail to the students, and an information sheet was sent home to their parents. Because the parents of government schools were better versed in local languages such as Hindi and Marathi, the information and consent forms were translated in these local languages. A total of 527 participants whose parents returned the signed consent forms and who provided written assent comprised the final list of eligible participants for the study. Permission to conduct the study at the school or college premise was sought from the Education Officer at BrihanMumbai Municipal Corporation (BMC) for government schools and from the principals of the selected private schools and junior colleges. The details of the sample selection are given in Figure 1. Participants completed the survey and anthropometry measurements during school/college hours under the supervision of the study investigators and class teachers. Ethics approval was obtained prior to the start of the study.

### 2.1 Sample size and sampling

To calculate the adequate sample size, we used the formula, *n* = *Z* ^2^ *P* (1 − *P*) divided by *d* ^2^; where *n* is the sample size, *Z* corresponds to the level of confidence, *P* is expected prevalence (as obtained from similar studies), and *d* indicates precision (Metcalfe, 2001).

Based on previous studies that had been conducted to assess the prevalence of depression among Indian adolescents (Aradhya, 2013; Jayanthi, Thirunavukarasu, & Rajkumar, 2015; Victor & Karunakaran, 2018), we considered the prevalence of depression among adolescents to be 20%. Using a 95% confidence interval (CI) and 5% precision, the estimated sample size was calculated to be 246. In order to get representation from southern and northern parts of the city, we estimated 492 participants. Considering a nonresponse rate of 10%, similar to previous studies, the final sample size was estimated as 541.

As per the study design, a total of 800 adolescents were eligible to participate; 273 adolescents were excluded from the study either because their parents did not return the signed consent forms (*n* = 186) or because the participants were not present on the survey day (*n* = 21) or had submitted incomplete information in the questionnaires (*n* = 66). The final sample included 527 adolescent girls and boys, ages 10–17 years, who were studying in Grade 5 to Grade 9 of three private schools and three government schools and Grade 11 of two junior colleges in Mumbai city.

### 2.2 Survey instrument

Participants completed a self‐administered survey instrument, which included questions related to depression symptoms, eating habits, sleep pattern and sociodemographic characteristics. Depression was assessed using Patient Health Questionnaire modified for Adolescents (PHQ‐A), that was developed by J Johnson in 2002. PHQ‐A is identical to the widely used and validated Patient Health Questionnaire (PHQ‐9), except a few minimal adjustments to incorporate age appropriate language‐ and depression‐related characteristics in adolescents such as feeling ‘irritable’ in the item assessing ‘feeling down, depressed’ and ‘weight loss’ to the question assessing ‘appetite’. PHQ‐A is available for use in the public domain (Johnson, Harris, Spitzer, & Williams, 2002). Though no psychometric data are available for PHQ‐A, given the similarity to PHQ‐9, the developers indicate using PHQ‐9 cutoffs to screen depression in adolescents. Several studies have validated the use of PHQ‐9 as an efficient tool for early detection and determination of the severity of depression in adolescents (Ganguly et al., 2013; Lewandowski et al., 2016; Richardson et al., 2010; M. M. Singh et al., 2017). We used the Hindi version of PHQ‐A for the participants attending government school and the English version was used for participants attending private schools and colleges. PHQ‐A comprised nine questions that measure the frequency of specific depression‐related symptoms during the past 2 weeks. Frequency response options are ‘not at all’, ‘several days’, ‘half of the days’ and ‘nearly daily’ and four dichotomous ‘severity’ questions that can be answered as ‘yes or no’. The scores can then be added to derive a combined severity score. Out of a total possible score of 27, depression scores ≥10 indicate positive for presence of depression (Ganguly et al., 2013; Lewandowski et al., 2016; K. Singh, Bassi, Junnarkar, & Negri, 2015; M. M. Singh et al., 2017). PHQ‐A scores can also be classified as minimal (PHQ‐A scores ≤4), mild (PHQ‐A scores 5–9), moderate (PHQ‐A scores 10–14), moderately severe (PHQ‐A scores 15–19) and severe depression (PHQ‐A scores 20–27).

Assessment of sleep patterns included questions on key sleep domains such as sleep schedule regularity (bedtime and wake up time), sleep quantity (frequency of ‘sleeping less than 6 hours’ and ‘sleeping more than 7 hours’ every night), daytime sleepiness (‘feel tired after waking up’) and sleep‐related problems (‘trouble falling asleep or sleeping too much’). The adolescents were asked to recall sleep patterns over a ‘typical’ recent week; the items were rated on a five‐point scale of *almost every day* if the sleep pattern was observed 4–6 times a week, *usually* if the pattern was observed 2–3 times a week, *sometimes* if observed once a week, *rarely* if observed for less than once a week and *almost never* if the sleep pattern never occurred. All of the questions included in the sleep survey were taken from questionnaires, such as the Pittsburgh Sleep Quality Index (Buysse, Reynolds, Monk, Berman, & Kupfer, 1989), Children's Sleep Habit Questionnaire (J. A. Owens, Spirito, & McGuinn, 2000) and Sleep Habits Survey (Wolfson et al., 2003), that have been used to examine sleep patterns in adolescents.

Eating habits related questions were drawn from a validated survey that we developed in our previous work (*Development and Evaluation of a Nutrition Transition ‐ Food Frequency Questionnaire for Adolescents in Western India*, unpublished) and included questions on the number of meals consumed per day and the weekly frequency of eating breakfast, eating meals with family at home and eating out with friends and family. The responses for the eating habit related questions included ‘everyday’, ‘3‐4 times a week’, ‘1‐2 days a week’, ‘less than once a week’ and ‘never’. The questionnaire also included demographic characteristics such as age, gender, number of family members, type of family (nuclear, joint, extended or single parent), religion and the working status of mother.

Prior to data collection, the survey instrument comprising PHQ‐A and questions related to eating habits, sleep patterns and demographic characteristics were pretested among 32 adolescents (12 participants attending a government school, 10 participants attending a private school and 10 participants attending a junior college in Mumbai), ages 10–17 years. All questions in the PHQ‐A were well understood, and no modifications were suggested. For the eating habit and sleep pattern‐related items, the participant responses were noted, and the questions that required clarifications were revised in consultation with field experts. The modified survey instrument was pilot tested among 10 adolescents selected from a private school and a government school before final administration. The schools and the junior college, where the pretesting and pilot testing were conducted were excluded from the main survey. The final survey was administered in English or Hindi to the study participants at their respective study sites under the supervision of trained and qualified research staff. Various measures such as regular data quality checks and back translation of the Hindi questionnaire into an English language questionnaire were taken in order to ensure the quality of the questionnaire. All research assistants received systematic training for administering the study protocol, assent/consent forms and survey and for collecting anthropometric measurements.

Weight, height, waist circumference and hip circumference were taken using standard methods (World Health Organization, 2008) on survey day. Standing height was measured using a portable stadiometer in centimetres and rounded to the nearest 0.1 cm, weight was measured using the TANITA electronic weighing scale in kilograms and rounded to the nearest 100 g, hip circumference was measured in centimetres and rounded to the nearest 0.1 cm and waist circumference was measured in centimetres and rounded to the nearest 0.1 cm using a calibrated measuring tape.

### 2.3 Statistical analyses

Statistical analysis was performed using SPSS version 22 (SPSS, Chicago, USA). BMI was calculated by dividing weight in kilogram by the square of height in meters. The *z* score values for weight for age, height for age and BMI for age were computed using WHO Anthro Software version 3.2.2 (‘WHO|Application tools’, 2018). Underweight was defined as BMI for age *z* scores below 2 standard deviation (SD), normal weight as *z* scores between −2SD and +1SD, overweight as above +1SD and obese as above +2SD (‘WHO|BMI‐for‐age (5‐19 years)’, 2019). Descriptive statistics such as frequency distribution for categorical variables and mean and SD for continuous variables and inferential statistics such as Student's *t* test, *χ* ^2^ test, odds ratio (OR) and regression analysis were used. Multivariate logistic regression analyses were used to analyse the association between multiple factors and depression. From bivariate analysis, risk factors significantly associated with the presence of depression were entered into a multivariate logistic regression model. Depression was considered the outcome (dependent variable) and dichotomized as none or mild depression (PHQ‐A 0–9) and moderate or severe depression (PHQ –A ≥ 10). Age (≥15 and <15 years), gender, sleep duration (sleep <6 h or ≥7 h per night) and sleep quality (feeling tired after waking up and trouble falling asleep/sleeping too much), type of institute attended (private schools, government schools and junior colleges), eating habits (skipping breakfast, having meals with family and eating out with friends) and body weight status (underweight, normal weight and overweight/obese) were entered as predictor (independent) variables. Adjusted ORs with 95% CI of OR were calculated, and *p* value ≤0.05 was set as significant.

### 2.4 Ethical considerations

Ethical approval was obtained from Intersystem Biomedica Ethics Committee, Mumbai, India.

## 3 RESULTS

Overall, 58% of the participants were girls, 32% were studying in government schools, 71% followed Hindu religion, 64% lived in nuclear families and 69% reported that their mothers were not working outside home. The characteristics of participants are presented in Table 1.

**TABLE 1:** Characteristics of study participants based on presence and severity of depression

Characteristics	Overall (*n* = 527)	Minimal depression (*n* = 57)	Mild depression (*n* = 338)	Moderate/severe depression (*n* = 132)	*p* value
Sociodemographic characteristics *n* (%)					
Gender					
Boys	223 (42.3)	24 (42.1)	154 (45.6)	45 (34.1)	0.545
Girls	304 (57.7)	33 (57.9)	184 (54.4)	87 (65.9)	
Age categories					
10–12 years	266 (50.5)	32 (56.1)	192 (56.8)	42 (31.8)	0.002^*^
13–15 years	153 (29.0)	21 (36.8)	97 (28.7)	35 (26.5)	
16–17 years	108 (20.5)	4 (7.0)	49 (14.5)	55 (41.7)	
Type of institution attended					
Government schools	170 (32.3)	15 (26.3)	114 (33.7)	41 (31.1)	≤0.001^**^
Private schools	249 (47.2)	36 (63.2)	176 (52.1)	37 (28.0)	
Junior colleges	108 (20.5)	6 (10.5)	48 (14.2)	54 (40.9)	
Type of living arrangement					
Single‐parent family	14 (2.7)	7 (12.3)	4 (1.2)	3 (2.3)	0.624
Nuclear family	336 (63.8)	18 (31.6)	236 (69.8)	82 (62.1)	
Joint family^a^	112 (21.3)	13 (22.8)	58 (17.2)	41 (31.1)	
Extended family^b^	65 (12.4)	19 (33.3)	40(11.8)	6 (4.5)	
Mothers' employment status					
Works full time(≥6 h/day)	108 (20.5)	30 (52.6)	47 (13.9)	31 (28.0)	0.321
Works part time(<5 h/day)	21(4.0)	8 (14.0)	10 (3.0)	3 (2.3)	
Does not work outside home	364 (69.1)	19 (33.3)	253 (74.9)	92 (69.7)	
Does not know/no response	34 (6.5)	0 (0.0)	28 (8.3)	6 (4.5)	
Religion					
Hindu	375 (71.2)	33(57.9)	280 (82.8)	62 (47.0)	0.762
Muslim	102 (19.3)	18 (31.6)	41(12.1)	43 (32.6)	
Christian	18 (3.4)	3 (5.3)	5 (1.5)	10 (7.6)	
Others (Jain, Sikh, Parsi, Buddhist)	32 (6.1)	3 (5.3)	12 (3.6)	17 (12.9)	
Body weight status *n* (%)					
Body mass index^c^					
Underweight	42 (8.0)	13 (22.5)	22 (6.5)	7 (5.3)	0.052^*^
Normal weight	246 (46.7)	28 (49.1)	165 (48.8)	53 (40.2)	
Overweight	125 (23.7)	7 (12.3)	78 (23.1)	40 (30.3)	
Obese	114 (21.6)	9 (15.8)	73 (21.6)	32 (24.2)	
Eating habit characteristics *n* (%)					
Skips breakfast > thrice a week	189 (35.9)	22 (38.6)	108 (32.0)	59 (44.7)	0.887
Skips breakfast ≤ twice a week	338 (64.1)	35 (61.4)	230 (68.0)	73 (53.3)	
Family meals > twice a week	160 (30.4)	25 (43.9)	103 (30.5)	32 (24.2)	0.034^*^
Family meals ≤ once a week	367 (69.6)	32 (56.1)	235 (69.5)	100 (75.8)	
Eats out with friends > thrice a week	157 (29.8)	10 (17.5)	73 (21.6)	74 (56.1)	0.297
Eats out with friends ≤ twice a week	370 (70.2)	47 (82.5)	265 (78.4)	58 (43.9)	
Sleep quantity *n* (%)					
≥7 h more than thrice a week	287 (54.5)	42 (73.7)	191 (56.5)	54 (40.9)	<0.001^**^
<6 h more than thrice a week	240 (45.5)	15 (26.3)	147 (43.5)	78 (59.1)	
Sleep quality *n* (%)					
Feels tired/sleep more than thrice a week	255 (48.4)	23 (40.4)	188 (55.6)	44 (33.3)	0.081
Trouble falling sleep more than thrice a week	272 (51.6)	34 (59.6)	150 (44.4)	88 (66.7)	

*Note:* Values are number and percentages; *p* values indicate level of significance for difference in characteristics between participants reporting minimal depression (Patient Health Questionnaire modified for Adolescents [PHQ‐A] scores ≤4), mild (PHQ‐A scores 5–9) and moderate to severe depression (PHQ‐A scores ≥10).

a Joint family indicates family where grandparents stay with parents of the participants in the same household.

b Extended family indicates where uncles/aunts/cousins stay together. Mean depression score using PHQ‐A was 7.9 ± 2.5.

c Body mass index (BMI) for age *z* scores are used to classify body weight status as underweight, normal weight, overweight and obese using World Health Organization (WHO) BMI for age cutoffs for 5–19 years.

** *p* value ≤0.05.

** *p* value ≤0.001.

The mean weight was 47.7 ± 15.3 kg (95% CI [46.3, 49.0]), mean height was 153 ± 10.3 cm (95% CI [152.1, 153.8]) and the mean BMI was 20.1 ± 4.9 kg/m^2^ (95% CI [19.7, 20.5]). The prevalence of underweight, normal weight, overweight and obesity was observed to be 8%, 46%, 24% and 22%, respectively, using BMI for age *z* scores (WHO, 2019). No significant gender differences were observed in the body weight status of the adolescents.

The mean number of daily meals consumed by the study participants was 3.3 ± 1.2; no significant difference was observed in the number of meals consumed between girls and boys, between early, middle and late adolescents and between participants from private schools, government schools and junior colleges. Overall, 55% reported eating breakfast ‘everyday’, 36% were having breakfast either ‘3‐4 times a week’, ‘1‐2 times a week’ or ‘less than once a week’ and 9% were not eating breakfast at all. While 22% reported sitting together with family for at least one meal ‘every day’, 61% were having meals with family either ‘3‐4 times a week’ or ‘1–2 times a week’ and 8% and 9% reported the frequency of family meals to be ‘less than once a week’ and ‘never’, respectively. A significantly higher percentage of girls reported sitting with family for meals as compared with the boys (*p* value <0.05).

**FIGURE 2:**
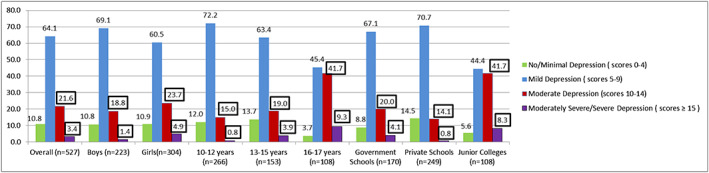
Overall prevalence of depression and between gender, age categories and type of institutes attended among 10‐ to17‐year‐old adolescents

The prevalence of minimal depression (PHQ‐A scores <5) was 11% and that of mild depression (PHQ‐A scores 5–9), moderate or moderately severe depression (PHQ‐ A scores 10–19) and severe depression (PHQ‐A scores >20) were observed to be 64%, 23.5% and 1.5%, respectively. The overall prevalence of depression and between genders, age categories and type of institutes attended are given in Figure 2.

A significantly higher prevalence of moderate to severe depression was observed among older adolescents, ages 16–17 years, and those attending junior college as compared with younger adolescents, ages 10–15 years, and studying in the schools (*p* value ≤0.05). No significant differences were observed in the prevalence of depression between genders, type of schools attended, living arrangements and mother's working status. Adolescents with moderately severe depression had a significantly higher body weight (67.6 ± 24.8 kg) and BMI (26.2 ± 6.6 kg/m^2^) than those with minimal depression (body weight 48.4 ± 13.1 kg; BMI was 20.2 ± 4.8 kg/m^2^) and mild depression (mean weight 47.2 ± 15.2 kg; mean BMI was 20.0 ± 4.8 kg/m^2^; *p* ≤ 0.05). Adolescents who reported sitting with family for meals once or less than once a week were observed to have significantly higher severity of depression than those who reported having family meals more than once a week (*p* value ≤0.05).

Depression symptoms and sleep patterns of 10‐ to 17‐year‐old adolescents in the study are presented in Table S1. More than half of the participants, 52% and 60%, reported feeling down/depressed and having little pleasure/interest in doing things at least ‘several days’ respectively in the past 2 weeks. One third reported feeling tired ‘half the days’ and having trouble concentrating ‘several days’ while 10% had trouble sleeping or were sleeping too much ‘almost every day’. Seventy‐seven percent reported feeling depressed or sad ‘most days’, even if they felt okay; 24% and 10% found that these problems had made it ‘somewhat difficult’ and ‘very difficult’ respectively for them to do their work, take care of things at home or get along with other people. Assessment of sleep habits indicated that 25% were feeling tired or sleepy and 14% had trouble falling asleep or were sleeping too much ‘almost every day’, respectively. Around 32% reported ‘sometimes’ sleeping for 7 h or more and two thirds were feeling sleepy and tired ‘at least once a week’.

The association between sleep patterns and depression symptoms was assessed. We observed a significant association between adolescents who reported experiencing depression symptoms such as feeling bad about themselves, having trouble concentrating throughout the day and being restless or too slow ‘several days’ in the PHQ‐A questionnaire and those who reported sleeping for less than 6 h, feeling tired/sleepy and having trouble falling asleep more than thrice a week in the sleep‐pattern‐related questions (*p* value ≤0.05).

In the multivariate regression analysis, the odds of having depression (PHQ‐A ≥ 10) was 4.5 times higher in adolescents who were having family meals less than once a week, 1.6 times higher among adolescents who were sleeping less than 6 h every night and 2.3 times higher among adolescents who reported having trouble falling asleep more than thrice a week (Table 2).

**TABLE 2:** Multivariate analysis of factors associated with depression (Patient Health Questionnaire modified for Adolescents [PHQ‐A] scores ≥10)

Characteristics	OR values	(95% CI)	*p* value
Age			
≥15 years	0.542	[0.268, 1.095]	0.088
Gender			
Boys	1.033	[0.543, 1.963]	0.922
Eating habits			
Skips breakfast more than thrice a week	0.942	[0.416, 2.130]	0.885
Family meals together once or less than once a week	4.577	[1.540, 8.602]	0.006^*^
Eats out with friends once or less than once a week	1.437	[0.497, 4.159]	0.504
Sleep quantity			
≥7 h more than thrice a week	0.750	[0.396, 1.418]	0.376
≤6 h more than thrice a week	1.674	[1.333, 1.862]	0.027^*^
Sleep quality			
Feels tired/sleep more than thrice a week	1.495	[0.789, 2.833]	0.217
Trouble falling sleep more than thrice a week	2.289	[1.853, 2.545]	≤ 0.001^**^
Body weight status			
Underweight	1.221	[0.422, 3.537]	0.712
Normal weight	1.400	[0.439, 4.462]	0.570
Overweight and obese	1.287	[0.343, 4.835]	0.708

Abbreviations: OR, to odds ratio; CI, confidence interval.

* *p* value significant at ≤0.05.

** *p* value significant at ≤0.001.

## 4 DISCUSSION

This study sought to determine the prevalence of depression and its association with eating habits, sleep patterns and body weight status among a convenience sample of 10‐ to 17‐year‐old adolescents in Mumbai, western India. Among this sample, 25% had moderate to severe depression (PHQ‐A ≥ 10) and 64% had mild depression (PHQ‐A 5–9). In general, the prevalence of depression has been reported to range from 10% to 68% among adolescents in India. The wide variations in prevalence rates can be attributed to the use of various screening tools for depression such as Beck's Depression Inventory (BDI) (Daryanavard et al., 2011; Jayanthi & Thirunavukarasu, 2015; Jha et al., 2017), Children Depression Rating Scale (CDRS) (Basker, Moses, Russell, & Russell, 2007) and General Health Questionnaire‐12 (GDQ‐12) (Goyal, Srivastava, & Bansal, 2009), different age categories of study participants and study settings such as rural or urban (Aradhya, 2013; Basker et al., 2007; Jayanthi & Thirunavukarasu, 2015; Jha et al., 2017; M. M. Singh, Gupta, & Grover, 2017). We used PHQ‐A to assess depression symptoms as this tool is specifically developed for the adolescent population and includes questions that are based on the Diagnostic and Statistical Manual of Mental Disorders (DSM) IV criteria for depression. Though PHQ‐A had been used as a screening measure of depression among adolescents in western settings (Lewandowski et al., 2016), there was no data on the use of PHQ‐A in Indian adolescents. Given that PHQ‐A uses items and cutoffs similar to those used in the PHQ‐9, we compared the results of this study with previous studies that had reported the prevalence of depression in adolescents in India using PHQ‐9. Similar to our findings, these studies have reported the prevalence of moderate to severe depression as 20% among 13‐ to 18‐year‐old adolescents (M. M. Singh et al., 2017) and as 21% in 15‐ to 19‐year‐old adolescents (Victor & Karunakaran, 2018).

In this study, the prevalence of depression was compared between older adolescents, ages 16–17 years, and early and middle adolescents, ages >15 years. We observed a high prevalence of moderate to severe depression among 10‐ to 12‐year‐old adolescents (32%) and among 13‐ to 15‐year‐old adolescents (27%) and that the prevalence of depression was significantly higher among older adolescents, ages 16–17 years, as compared with early and middle adolescents, ages >15 years. Similar findings were reported in a study that was conducted among11‐ to 15‐year‐old adolescents (*n* = 9863) in the United States (Saluja et al., 2004). Several factors may act as emotional stressors and contribute to a higher prevalence of depressive symptoms among older adolescents such as changing relationships with peers and parents, the desire for autonomy, an increase in academic pressure, the need to fit in a social environment and risk‐taking behaviours such as episodic drinking and smoking (Maughan et al., 2013; K. Singh et al., 2015; Thapar et al., 2012). The finding that a relatively high proportion of early and middle adolescents had depression highlights a need to identify and intervene as early as possible to manage depression more effectively and prevent future depression‐related negative outcomes.

Besides evaluating prevalence and severity of depression symptoms, this study looked at the sleep patterns and eating habits of adolescents. The results showed that a high proportion of adolescents were sleep deprived and that sleep insufficiency was associated with depression symptoms among participants. Short sleep duration and poor sleep quality results in daytime sleepiness and feeling tired throughout the day, which interferes with alertness, academic performance and social interactions, thus increasing the risk of developing depressive symptoms among adolescents (American Academy of Pediatrics Supports Childhood Sleep Guidelines, n.d.; Chaput et al., 2016; Owens et al., 2014). Few studies that assessed the role of sleep in adolescent depression reported that sleep‐disturbed participants were more likely to be depressed and had more severe depressive symptoms as compared with adolescents who did not experience sleep disturbances (Alvaro et al., 2013; Baglioni et al., 2011). In this study, it was observed that the participants who reported feeling down, feeling bad about themselves and having trouble concentrating several days in the past fortnight (as reported in PHQ‐A) were sleeping for less than 6 h at least thrice a week (*p* value ≤0.05), indicating a possible association between sleep patterns and depression symptoms. Studies from various parts of the world have reported similar associations between sleep patterns and depression in adolescents (Alvaro et al., 2013; Baglioni et al., 2011; Hyong et al., 2008; Liu et al., 2007).

Among the eating habits that were evaluated in this study, we observed a significant inverse association between the severity of depression and the frequency of having meals together as a family (OR 4.58; 95% CI [1.54, 13.60]; *p* ≤ 0.05). The finding is consistent with a previous study that evaluated the correlation between family meals and the psychosocial well‐being of adolescents and reported that having regular family meals may improve family connectedness and depressive symptoms (Eisenberg, Olson, Neumark‐Sztainer, Story, & Bearinger, 2004). Having regular family meals fosters healthier dietary practices (Gillman et al., 2000) and can also work as a protective factor towards reducing the risk of developing depression in adolescents (Kim, Lee, Suh, & Kim, 2013). Considering the impact that family structure, interpersonal relationships and a supportive family environment may have on emotional and mental health of an adolescent (Behere, Basnet, & Campbell, 2017; Chen et al., 2017; Yu et al., 2015), encouraging family meals present a convenient avenue to build better lifestyle behaviours, enhance family bonding and also improve the socio‐emotional health of adolescents.

The relationship between body weight status and depression was also assessed in the study. We observed that adolescents with moderate to severe depression had significantly higher BMI than those with minimal depression. Evidence regarding association between depression and body weight status is complex. While a few studies have shown a linear association (or an increase in weight predicts higher depression) between BMI and depression (Eidsdottir, Kristjansson, Sigfusdottir, Garber, & Allegrante, 2014; Jorm et al., 2003; Needham & Crosnoe, 2005), others have reported a U‐shaped trend (both underweight and obesity are associated with depression) between depression and BMI (De Wit, Van Straten, Van Herten, Penninx, & Cuijpers, 2009). Nevertheless, available data suggest that unhealthy body weight status may contribute to body dissatisfaction and decreased self‐esteem (Choi & Choi, 2016; Murray, Rieger, & Byrne, 2018; Reel, Voelker, & Greenleaf, 2015), which may become a risk factor for psychological distress and depression among adolescents. Thus, it is important that approaches that encourage healthy weight status and a positive body image are integrated with those that aim at alleviating depression among adolescents.

Briefly, to our knowledge, this study is the first to assess and compare the prevalence of depression and severity of symptoms among the entire spectrum of adolescence ages 10–17 years in a major metropolitan city in India. In addition, given that little is known about adolescents in such rapidly developing regions, we sought to investigate the relationship between depression, sleep, eating habits and body weight status among this population. We assessed depression using the PHQ‐A and a validated diet questionnaire that we had developed in previous work. However, there are a few limitations to this study. First, similar to all cross‐sectional studies, any causal relationships or temporal associations between variables cannot be established and will require further studies to examine associations between depression and sleep and eating habits in adolescents. Second, there can be potential confounder variables related to lifestyle behaviours such as smoking, alcohol abuse and physical inactivity that may not have been adequately controlled and may have impacted the results, though several studies that have adjusted for lifestyle behaviours in many ways have consistently elaborated a positive association between sleep and depression (Jacka et al., 2011; Roberts & Duong, 2014) and an inverse relation between diet and depression (Bamber et al., 2007; Lang et al., 2015; Quirk et al., 2013; Wattick et al., 2018). Self‐report bias and lack of more subjective measures like semiquantitative food frequency questionnaires for dietary assessments must also be considered in future studies.

## 5 CONCLUSION

The study showed that 25% of the adolescents, ages 10–17 years, attending select schools and colleges in Mumbai, India, had moderate to severe depression; the depressive symptoms were prevalent, after accounting for gender, age, socioeconomic status (SES; type of school attended was used as a proxy for SES) and body weight status. Additionally, the adolescents in the study reported poor eating habits and sleep insufficiency—persistence of these problems may lead to adverse health consequences. A significant association between depression and sleep duration (sleeping less than 6 hours a day) and sleep quality (having trouble falling to sleep/sleeping too much) was observed, accentuating a need to address sleep disturbances and depression symptoms together among adolescents. As such, sleep deprivation is a matter of growing concern with consequences tracking into adulthood, in terms of both physical health and mental health; the association between sleep insufficiency, sleep disturbances and depression demands further research and concerted interventions so as to inform recommendations for improving sleep and preventing depression among adolescents in India. The finding that the prevalence of depression was lower in households where families ate meals together suggests the potential role of family mealtimes and supportive family environments to foster healthy eating habits and improve mental health of adolescents. Early identification of depression is critical but so are efforts to improve modifiable lifestyle behaviours such as sleep and dietary patterns. Administration of simple tools for screening depression and timely diagnosis may aid in effective care for adolescents with depression and evaluating sleep and eating habits of adolescents might generate additional benefits.

## CONFLICTS OF INTEREST

The authors declare no potential conflict of interests.

## CONTRIBUTIONS

PM and JM formulated the research questions and designed the study; PM supervised field work and data management. JM and PM analysed the data, and PM drafted the manuscript with inputs received from NIS. The final manuscript was revised and approved by all authors.

## Supporting information

Table S1: Depression Symptoms and Sleep Patterns of 10–17 year old Adolescents in the Study (*n* = 527)
