# Supplementary material for: Eating habits and sleep patterns of adolescents with depression symptoms in Mumbai, India
Source: Matern Child Nutr. 2020 Dec 21;16(Suppl 3):e12998. doi: 10.1111/mcn.12998 (PMC7752132; doi:10.1111/mcn.12998)
Supplement: Supplementary file 1 — Table S1: Depression Symptoms and Sleep Patterns of 10–17 year old Adolescents in the Study (n = 527) [file MCN-16-e12998-s001.docx]

**Supplementary Table 1: Depression Symptoms and Sleep Patterns of 10-17 year old Adolescents in the Study (n =527)**

| ***Depression Symptoms*** | **Frequency of experiencing depression symptoms in**  **past two weeks** | | | | |
| --- | --- | --- | --- | --- | --- |
|  | **Not at all** | **Several Days** | | **Half of the days** | **Nearly daily** |
| Feeling down, depressed | 230 (43.6) | 158 (30.0) | | 117 (22.2) | 22 (4.2) |
| Little interest/pleasure in doing things | 146 (27.7) | 165 (31.3) | | 148 (28.1) | 68 (12.9) |
| Trouble Falling Asleep/ Sleeping too much | 171(32.4) | 169 (32.1) | | 132 (25.0) | 55 (10.4) |
| Poor Appetite | 303 (57.5) | 153 (29.0) | | 60 (11.4) | 11(2.1) |
| Feel tired | 131(24.9) | 167 (31.7) | | 163 (30.9) | 66 (12.5) |
| Feel bad about yourself | 298 (56.5) | 160 (30.4) | | 60 (11.4) | 9 (1.7) |
| Trouble concentrating | 212 (40.2) | 180 (34.2) | | 106 (20.1) | 29 (5.5) |
| Being restless, too slow | 434 (82.4) | 75 (14.2) | | 17 (3.2) | 1 (0.2) |
| Thoughts of being dead | 484 (91.8) | 38 (7.2) | | 5 (0.9) | 0 (0) |
| ***Sleep Patterns*** | **Frequency of reported sleep quantity and sleep quality**  **in past week**† | | | | |
|  | **Rarely/**  **Almost Never** | | **Sometimes** | **Usually** | **Almost Everyday** |
| ***Sleep Quantity*** | | | | | |
| Sleep ≥ 7 hours | 69(13.1) | | 171(32.4) | 104(19.7) | 183(34.7) |
| ***Sleep Quality*** | | | | | |
| Feel tired/sleepy | 120(22.8) | | 152(28.8) | 121(23.0) | 134(25.4) |
| Trouble falling asleep/Sleeping too much | 178(33.8) | | 198(37.6) | 79(15.0) | 72(13.7) |

Data is presented as number and percentage in parentheses.

† ‘Almost every day’ indicates that the sleep pattern was observed 4-6 times, ‘usually’ indicates that the sleep pattern was observed 2-3 times, ‘sometimes’ indicates once a week, ‘rarely’/ almost never indicates less than once a week or never observed in past one week.
